# EMT-like circulating tumor cells in ovarian cancer patients are enriched by platinum-based chemotherapy

**DOI:** 10.18632/oncotarget.16179

**Published:** 2017-03-14

**Authors:** Issam Chebouti, Sabine Kasimir-Bauer, Paul Buderath, Pauline Wimberger, Siegfried Hauch, Rainer Kimmig, Jan Dominik Kuhlmann

**Affiliations:** ^1^ Department of Gynecology and Obstetrics, University Hospital Essen, 45147 Essen, Germany; ^2^ Department of Gynecology and Obstetrics, Medical Faculty and University Hospital Carl Gustav Carus, Technische Universität Dresden, 01307 Dresden, Germany; ^3^ National Center for Tumor Diseases (NCT), Partner Site Dresden, 69120 Heidelberg, Germany; ^4^ German Cancer Consortium (DKTK), Dresden and German Cancer Research Center (DKFZ), 69120 Heidelberg, Germany; ^5^ QIAGEN, 40724 Hilden, Germany

**Keywords:** ovarian cancer, circulating tumor cells, epithelial-to-mesenchymal-transition, PI3Kα, Akt-2

## Abstract

**Background:**

Assuming that tumor cell dissemination requires a shift to a mesenchymal phenotype, we analyzed the incidence of epithelial-to-mesenchymal-transition (EMT)-like circulating tumor cells (CTCs) in ovarian cancer patients and inquired, how their molecular phenotypes respond to platinum-based chemotherapy and influence outcome.

**Results:**

Before surgery, overall detection rate for epithelial CTCs was 18%. EMT-like CTCs were more frequently observed (30%) and were mutually exclusive to epithelial CTCs in the majority of patients (82%). After chemotherapy, EMT-like CTCs increased up to 52%, accompanied by the “de novo” emergence of PI3Kα+/Twist+ EMT-like CTCs. Before surgery, PI3K+ EMT-like CTCs in combination with epithelial CTCs indicated decreased OS (*p* = 0.02) and FIGO I-III patients with residual tumor burden after surgery were more likely to be positive for EMT-like CTCs after chemotherapy (*p* = 0.02). In the latter group, epithelial CTCs alone significantly correlated with decreased PFS and OS (*p* = 0.02, *p* = 0.002), supported by an additional inclusion of PI3K+ CTCs (OS, *p* = 0.001).

**Materials and Methods:**

Blood samples of 91 ovarian cancer patients before surgery and 31 matched samples after adjuvant chemotherapy were evaluated for CTCs with the AdnaTest *ovarian cancer* and *EMT-1*, analyzing the epithelial-associated transcripts EpCAM, Muc-1 and CA125 and the EMT-associated transcripts PI3Kα, Akt-2 and Twist.

**Conclusions:**

Platinum-based chemotherapy seems to select for EMT-like CTCs in ovarian cancer patients and provokes a shift towards PI3Kα and Twist expressing CTCs, which may reflect clonal tumor evolution towards therapy resistance. It has to be determined, whether this CTC subgroup may serve as a biomarker to identify patients at high risk.

## INTRODUCTION

Epithelial ovarian cancer is the fifth leading cause of cancer death among women in Europe and the United States and the second most common gynecological malignancy [1]. Most cases are diagnosed in advanced stages and although response rates to chemotherapy reach up to 80%, the majority of patients cannot be cured. Standard treatment of advanced ovarian cancer is primary surgery aiming at complete resection followed by platinum and paclitaxel based chemotherapy, which has been shown to prolong progression free survival (PFS) as well as overall survival (OS) [2]. Postoperative residual tumor is one of the most important prognostic factors in advanced ovarian cancer [3–5]. Meanwhile, although new multimodal therapeutic concepts, such as antiangiogenic therapy (e.g. Bevacizumab) or PARP-inhibition (only for recurrent patients), have been designed, more than half of all patients still experience recurrence, resulting in a poor overall prognosis [6–8]. Thus, the identification of innovative therapeutic targets and the identification of predicitive and prognostic biomarker concepts are highly desirable.

In this regard, circulating tumor cells (CTCs) in the blood and disseminated tumor cells (DTCs) in the bone marrow (BM) have already been shown to be promising candidates [9]. Despite the fact that CTCs indicate poor prognosis [10–14], we recently reported that excision repair cross-complementation group 1 protein (ERCC-1)-positive CTCs are present in 8% of patients and constitute an independent predictor, not only for OS but also for PFS. Most interestingly, we discovered the presence of ERCC1^+^CTC at primary diagnosis to be an independent predictor for platinum-resistance, whereas ERCC1-expression in corresponding primary tumor tissue predicted neither platinum-resistance nor prognosis [15]. Moreover, we reported that the presence of DTCs in the BM, as well as their persistence after platinum based chemotherapy, correlates with poor prognosis and is accompanied by stem cell characteristics of DTCs [16].

The broad heterogeneity of CTCs of cancer patients, including ovarian cancer, has already been demonstrated [17–19] and we may speculate that, besides the presence of epithelial, stem cell-like or potentially platinum-resistant ERCC1-expressing CTCs, some other CTC-phenotypes may play a dominant role for therapy resistance and recurrence in ovarian cancer patients. In this regard, it has been hypothesized that disseminating epithelial cancer cells may undergo a variety of biochemical changes and reversibly acquire fibroblastoid or mesenchymal traits, known as epithelial-to-mesenchymal-transition (EMT), which has already been described for breast cancer CTCs [20, 21]. EMT occurs under physiological conditions, however, is also a key mechanism for malignant progression. “Oncogenic EMT” allows tumor cells to acquire invasive properties and to develop metastatic growth characteristics. Moreover, it protects them from hostile conditions during the dissemination process. Interestingly, disseminated tumor cells may revert to their original epithelial phenotype, referred to as mesenchymal-epithelial-transition (MET), which promotes their colonization and contributes to the establishment of ultimately metastatic sites [22–29].

The PI3K/Akt/mTOR signalling pathway is aberrantly activated in the majority of human malignancies and confers oncogenic functions by promoting proliferation and cell survival [30, 31]. Therefore, this pathway has attracted widespread attention as therapeutic target for several malignancies [32]. Interestingly, recent evidence suggested that the PI3K/Akt/mTOR pathway is also essentially involved in EMT-regulation, thereby promoting tumor aggressiveness [33]. Moreover, repression of the cellular adhesion molecule E-cadherin, which is considered a hallmark of EMT, is mediated by EMT-associated transcription factors, such as Twist, Snail, Slug or Zeb [34]. Of those, especially Twist raised considerable attention, since it can also mediate invasiveness, drug resistance and EMT through a positive feedback loop with Akt, therefore providing a direct link to the PI3K/Akt/mTOR pathway [33].

In ovarian cancer, aberrant activation of the PI3K/Akt/mTOR pathway has also been reported and EMT is supposed to promote chemo-resistance [30, 35]. Interestingly, it has been suggested that cisplatin treatment of ovarian cancer cells generates residual cells with EMT-like traits [36]. However, it is still actively discussed, whether ovarian cancer cells in the primary tumor actually undergo a complete transition to a mesenchymal state [37]. Moreover, it is unknown, whether EMT-associated phenotypes extend to CTCs in the blood of ovarian cancer patients and whether they contribute to the heterogeneity of ovarian cancer CTCs. Therefore, assuming that dissemination of ovarian cancer cells requires at least a partial shift to a mesenchymal phenotype, the main purpose of our study was to analyze the incidence of epithelial and EMT-like CTCs at primary diagnosis of ovarian cancer. Moreover, we investigated how their detection rate is influenced by platinum-based chemotherapy. As a secondary objective, we analyzed EMT-associated transcript markers in more detail and were interested, how particular molecular phenotypes of EMT-like CTCs respond to platinum-based chemotherapy.

## RESULTS

### Platinum-based chemotherapy selects for EMT-like CTCs in ovarian cancer

**Figure 1:**
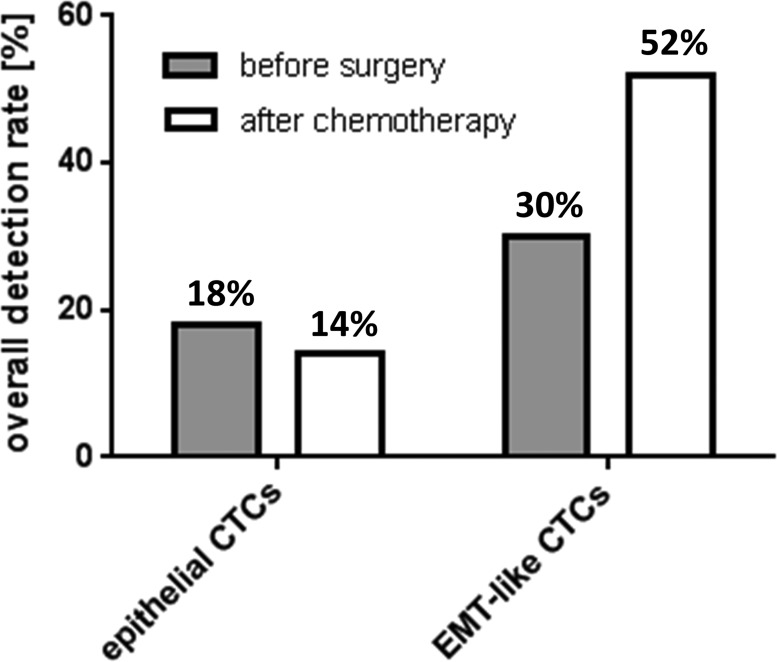
Overall detection frequency of epithelial and EMT-like CTCs in ovarian cancer The bar chart illustrates overall detection rates of epithelial and EMT-like CTCs in ovarian cancer patients before surgery and after chemotherapy. Percentages for the two classes of CTCs were calculated independently from each other and, in both cases, refer to the whole study population (before surgery: *n* = 91, after chemotherapy *n* = 31). A patient was considered “epithelial CTC-positive” or “EMT-like CTC-positive”, if at least one of the epithelial markers or one of the EMT-associated markers was detectable, respectively.

We analyzed the epithelial associated marker transcripts EpCAM, Muc-1 and CA-125 as well as the representative EMT-associated marker transcripts PI3Kα, Akt-2 and Twist before surgery (*n* = 91) and in paired blood-samples after platinum-based chemotherapy (*n* = 31). Positivity for each CTC-subtype was defined by the detection of at least one of the transcripts of each marker panel, respectively. Epithelial CTCs were detected with an overall incidence of 18% before surgery, which slightly decreased to 14% after platinum-based chemotherapy. EMT-like CTCs were observed with a considerably higher detection rate at baseline (30%) and their incidence further increased after chemotherapy to an overall detection frequency of 52% (Figure 1, Supplementary Table 1), suggesting that platinum-based chemotherapy selects for EMT-like CTCs.

### Epithelial and EMT-like CTCs exhibit a low phenotypic overlap

**Figure 2:**
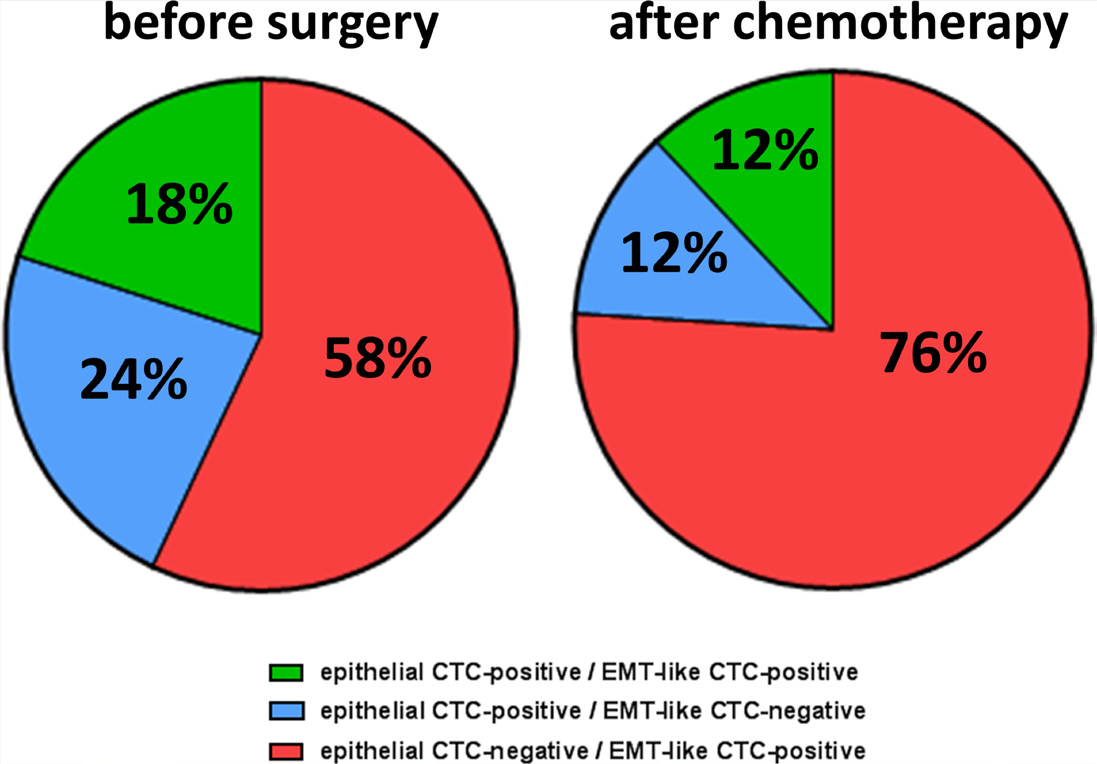
Phenotypic overlap of epithelial and EMT-like CTCs The pie chart depicts the overlap of epithelial and EMT-like CTCs in ovarian cancer patients before surgery and after chemotherapy. Percentages were calculated in reference to all patients with overall CTC-positivity. Besides patients with exclusively epithelial (blue) and exclusively EMT-like CTCs (red), there were also patients, harbouring both CTC populations in their blood (green).

Having described the incidence of epithelial and EMT-like CTCs by separate analyses, we now investigated the overlap between epithelial and EMT-associated phenotypes (Figure 2, Supplementary Table 1). Before surgery, patients with detectable CTCs were positive for either EMT-associated transcripts (58%) or epithelial-associated transcripts (24%). Thus, epithelial and EMT-like CTCs were mutually exclusive in the majority of patients (82% in total). Interestingly, only a minor fraction of patients showed up with dual positivity of epithelial and EMT-like CTCs (18%), indicating that epithelial- and EMT-like CTCs seem to represent mostly independent CTC populations with low phenotypic overlap. After chemotherapy, this trend was retained and the proportion of exclusively EMT-positive CTCs further increased up to 76%, whereas the number of patients with exclusively epithelial CTCs or dual positivity each declined to 12%.

### PI3Kα and Twist positive EMT-like CTCs are specifically enriched by platinum-based chemotherapy

**Figure 3:**
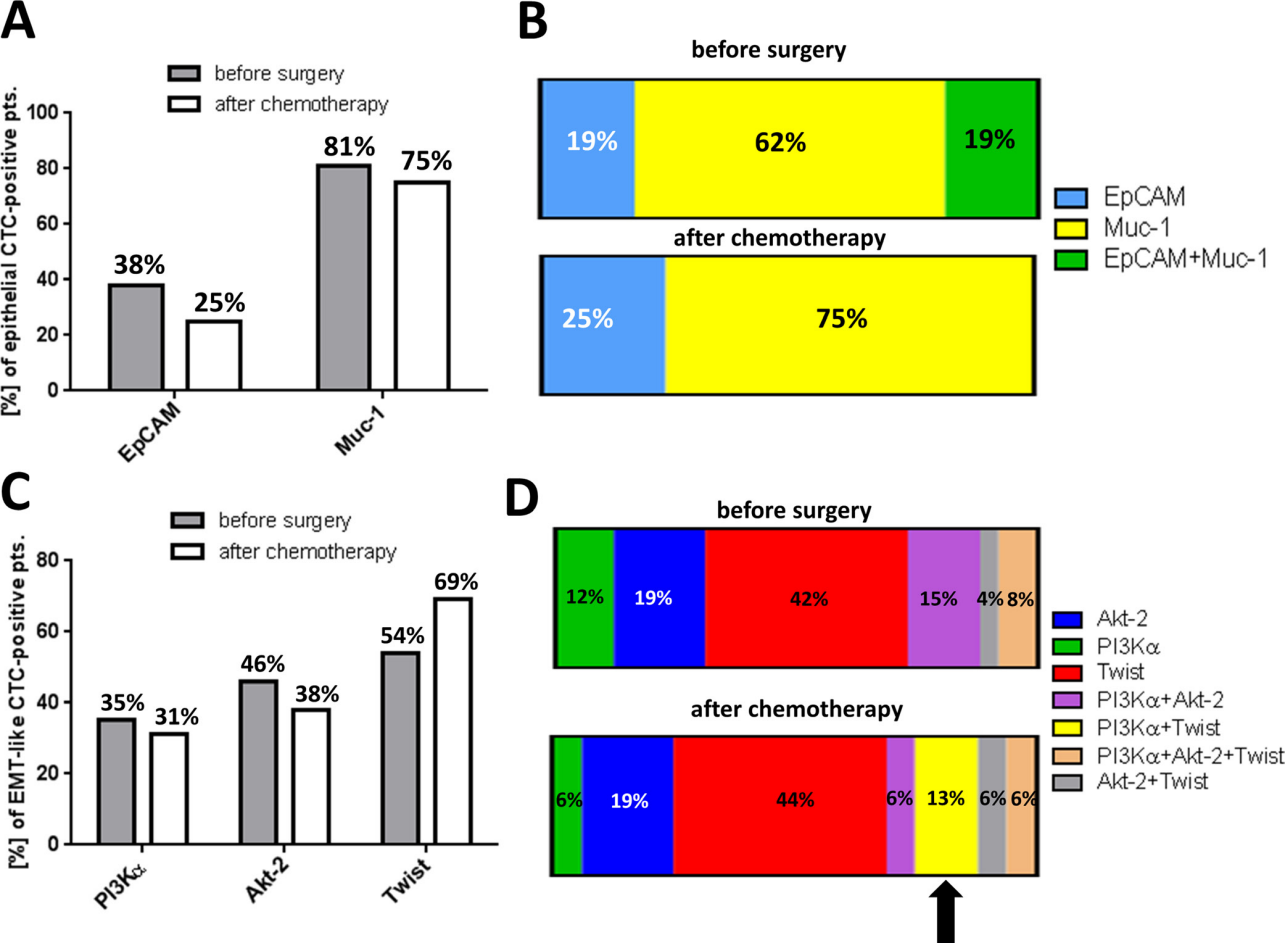
Heterogeneity of EMT-associated CTC-phenotypes and their response to platinum-based chemotherapy The figure summarizes molecular phenotypes of EMT-like CTCs and their response to platinum-based chemotherapy. (**A**) The bar chart shows the marker distribution in epithelial CTC-positive patients. Percentages for EpCAM and Muc-1 were calculated independently from each other and in reference to only those patients with positivity for epithelial CTCs. (**B**) The stacked bar chart illustrates the marker distribution of epithelial CTC-positive patients, also considering dual-positivity for EpCAM and Muc-1. (**C**) The bar chart shows the marker distribution in EMT-like CTC-positive patients. Percentages for PI3Kα, Akt-2 and Twist were calculated independently from each other and in reference to only those patients with positivity for EMT-like CTCs. (**D**) The stacked bar chart illustrates the marker distribution in EMT-like CTC-positive patients, now also considering dual- or triple positivity for EMT-associated transcripts.

We were further interested in the molecular phenotypes of epithelial and EMT-like CTCs in ovarian cancer and their response to platinum-based chemotherapy. Figure 3A illustrates the marker distribution among epithelial CTCs. Detection frequencies for EpCAM and Muc-1 were calculated independently from each other and in reference to only those patients with positivity for epithelial CTCs. Before surgery, positivity for epithelial CTCs mostly resulted from Muc-1 positivity, which was detected in 81% of cases, whereas EpCAM transcripts were less frequently detected (38%). After chemotherapy, detection frequency was slightly reduced for both marker transcripts; however, Muc-1 positivity remained three times more abundant than EpCAM-positivity (75% Muc-1-positive vs. 25% EpCAM-positive, Supplementary Table 1).

Figure 3B depicts the molecular heterogeneity of epithelial CTCs in more detail. Percentages were calculated in reference to only those patients with positivity for epithelial CTCs, now also considering dual positivity. Before surgery, the majority of epithelial CTCs were exclusively Muc-1 positive (62%), whereas only 19% were exclusively EpCAM-positive and further 19% showed dual positivity for Muc-1 and EpCAM. After chemotherapy, dual EpCAM+Muc-1-positive CTCs were no longer detectable and only Muc-1 (75%) or EpCAM (25%) positive CTCs were observed. CA-125 transcripts could not be detected at any time in our patient cohort, indicating expression levels below the detection limit of our assay or complete absence of CA-125 transcripts in the enriched CTC populations (Supplementary Table 1).

Subsequently, we performed the same kind of analysis for the EMT-associated marker panel (Figure 3C). Here again, percentages for PI3Kα, Akt-2 and Twist were calculated independently from each other in reference to only those patients with positivity for EMT-like CTCs. Before surgery, PI3Kα was observed in 35% of patients with positivity for EMT-like CTCs and Akt-2 in 46% of patients. Twist was most frequently detected (54%, Supplementary Table 1). Interestingly, after platinum-based chemotherapy, positivity rates for PI3Kα and Akt-2 slightly decreased, whereas Twist positivity was substantially elevated up to 69% in post-therapeutic blood samples, which is in accordance to the overall increase of EMT-like CTC (Figure 1).

Figure 3D depicts the molecular heterogeneity of EMT-like CTCs in more detail. Percentages were calculated in reference to only those patients with positivity for EMT-like CTCs, now also considering dual or triple positivity. Before surgery, exclusively Twist positive CTCs were most abundant (42%) followed by Akt-2 (19%) and PI3Kα-positive CTCs (12%). Additional CTC-phenotypes with dual or triple positivity for EMT-markers were observed with low or moderate detection frequency (15% PI3Kα+Akt-2 / 4% Akt-2+Twist / 8% PI3Kα+Akt-2+Twist). After chemotherapy, selective changes in the composition of molecular CTC-phenotypes became obvious. Interestingly, an additional molecular CTC-phenotype with dual PI3Kα+Twist positivity emerged, mostly, at the expense of PI3Kα+Akt-2-positive CTCs (Supplementary Table 1). The proportion of the other CTC-phenotypes remained largely stable in response to platinum-based chemotherapy. Notably, while the proportion of exclusively Twist or exclusively PI3Kα positive CTCs also remained nearly unchanged in post-chemotherapeutic blood samples, we conclude that the increase in the overall incidence of EMT-like CTCs (as shown in Figure 1) is accompanied by the “de novo” emergence of a dual PI3Kα+Twist positive CTCs after chemotherapy.

### Clinical relevance of EMT-like CTCs

We inquired, whether the presence and enrichment of EMT-like CTC subtypes correlates with the patient's clinicopathological parameters or with their survival. We observed the trend that patients with a residual tumor burden after primary debulking surgery were more likely to have EMT-like CTCs in their blood after adjuvant chemotherapy than patients with a macroscopically complete tumor resection. This association became statistically significant, after excluding patients with distant metastasis (FIGO IV; *p* = 0.02).

**Figure 4:**
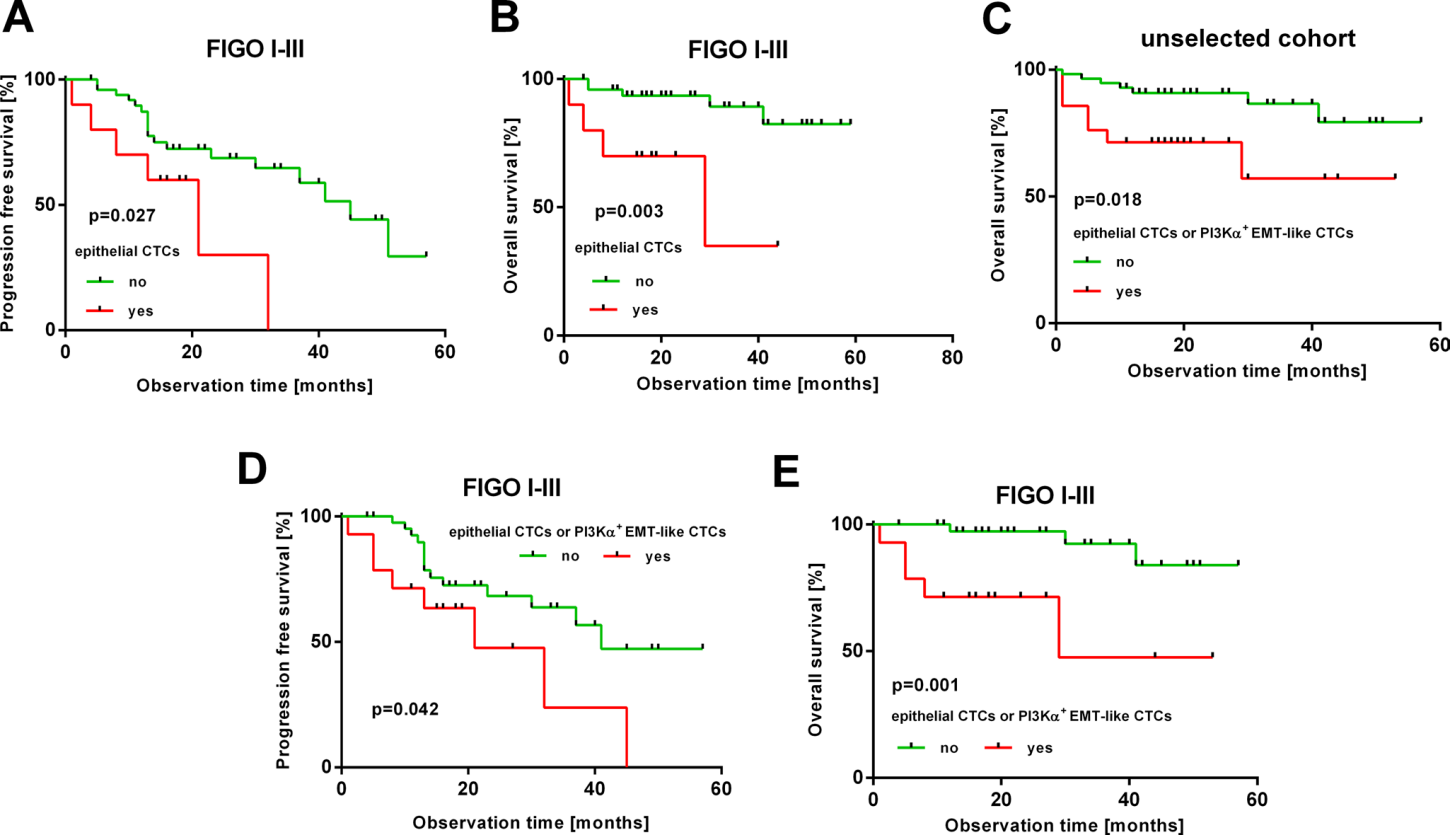
Prognostic relevance of epithelial and EMT-like CTCs The Kaplan-Meier plots show prognostic relevance of different CTC-subtypes at primary diagnosis. (**A, B**): epithelial CTCs in FIGO I-III patients (thus without distant metastasis) (**C**): epithelial or PI3Kα-positive CTCs in the unselected patient cohort (**D, E**): epithelial or PI3Kα-positive EMT-like CTCs in FIGO I-III patients. Red curves represent patients, positive for the respective CTC-subtype(s); green curves represent patients, negative for the indicated CTC-subtype(s).

Subsequently, we investigated prognostic significance of epithelial and EMT-like CTCs before surgery and after chemotherapy by Kaplan-Meier analysis. In the unselected total patient cohort, no prognostic relevance of epithelial CTCs before surgery or after chemotherapy could be demonstrated (Supplementary Figure 3). However, after excluding patients with FIGO IV as possible confounders, which per se have a poor prognosis, the presence of epithelial CTCs at primary diagnosis significantly indicated decreased PFS (HR: 2.63, 95% CI: 1.24–15.53; *p* = 0.027) and OS (HR: 5.79, 95% CI: 2.98–162.7; *p* = 0.003, Figure 4A, 4B). There was no prognostic significance of EMT-like CTCs before surgery or after chemotherapy in the total patient cohort (Supplementary Figure 4). However, combined analysis showed that the presence of epithelial CTCs or PI3Kα transcripts indicates reduced OS in the total study population (HR: 3.25, 95% CI: 1.31–15.47; *p* = 0.018, Figure 4C). Interestingly, this finding could be confirmed with increased statistical significance, after excluding FIGO IV patients, and the presence of epithelial CTCs or PI3Kα transcripts at primary diagnosis indicated reduced PFS (HR: 2.35, 95% CI: 1.06–8.74; *p* = 0.042) and OS (HR: 7.22, 95% CI: 3.21–111.5; *p* = 0.001, Figure 4D, 4E).

## DISCUSSION

In the present investigation, we analyzed incidence and molecular phenotypes of EMT-like CTCs in the blood of ovarian cancer patients and monitored their response to platinum-based chemotherapy. EMT-like CTCs were, already at primary diagnosis, more abundantly detected than epithelial CTCs and showed low phenotypic overlap with epithelial CTCs. After chemotherapy, we observed a selective enrichment of EMT-positive CTCs, which was accompanied by the “de novo” emergence of dual PI3Kα and Twist positive CTCs.

At present, there is no standard definition for the identification of CTCs and a variety of CTC-enrichment and detection strategies are available, based on CTC-associated surface antigens or intrinsic (physical or tumor-biological) CTC properties, such as size, deformability, invasive capacity or telomerase activity [38, 39]. Subsequent CTC-detection can be carried out by a broad spectrum of methods, such as immunocytological or molecular biology based assays [10, 11, 39–43]. In line with our previous investigations [10, 15], we took advantage of the AdnaTest *OvarianCancer*, which allows a more detailed molecular characterization of enriched CTCs. Notably, overall detection frequency of epithelial CTCs at primary diagnosis, reported herein, was comparable to our previous analyses, confirming comparability of the underlying study with our previous observations on larger patient cohorts [10]. In the present investigation, we were interested in the incidence and dynamics of EMT-associated CTC-phenotypes, which, to the best of our knowledge, has never been investigated in ovarian cancer patients so far.

We observed that EMT-associated marker transcripts considerably expanded the phenotypic range of CTC-detection. Already at primary diagnosis, EMT-like CTCs were more abundant than epithelial CTCs. This is in accordance with findings on breast cancer, reporting that EMT is a rare event in the primary tumor, however, frequently occurs among CTCs [21, 44]. Therefore, we conclude that EMT is also a common event among ovarian cancer CTCs and might already have been initiated in the primary tumor. Interestingly, recent data from a pancreatic cancer mouse model even suggested that EMT-like CTCs can already be shed into the circulation from pre-invasive lesions [45].

We explicitly used the term EMT-“like” CTCs in our study and strictly avoiding any other descriptive terms that might imply that these CTCs already acquired a fully mesenchymal state. Since the immunomagnetic enrichment of CTCs in our assay is based on the epithelial surface epitopes Muc and EpCAM, this assay cannot detect fully mesenchymal CTCs, which have completely downregulated their epithelial surface epitopes. Thus, EMT-like CTCs, selected and characterized herein, express the epithelial marker proteins EpCAM and Muc on their surface, allowing immunomagnetic selection, however, do not express EpCAM or Muc-1 on transcript level above the detection limit of our assay. At a first glance, this may appear counterintuitive, however, discordances between protein and transcript expression profiles of a cell can be explained by post-transcriptional modifications of messenger RNA or differences in the half-live time between messenger RNA and their corresponding proteins [46–48]. Notably, since these CTCs also co-express Akt-2, PI3Kα or Twist, we describe snapshots of “semi-mesenchymal” CTCs [49]. Semi-mesenchymal CTCs could be either on their way to an ultimately mesenchymal phenotype (EMT) or on their way back to an epithelial phenotype (MET) or, alternatively, they could persist in this intermediate state. Although the biology of semi-mesenchymal CTCs is largely unknown, we could speculate that particularly a semi-mesenchymal state reflects an aggressive CTC-phenotype with high degree of plasticity, which facilitates the adaption of CTCs to hostile environmental stimuli during dissemination. This is in line with the hypothesis, that EMT (and its reversion MET) is a highly dynamic process and describes different continuous phenotypes, rather than a dichotomous switch between epithelial and mesenchymal states. Notably, those phenotypic changes directly influence the yield of CTC-detection assays that are based on epithelial selection markers [49]. However, our assumption of continuous CTC phenotypes in ovarian cancer is not necessarily supported by the fact that we observed a trend for a mutual exclusion between patients with only epithelial CTCs and those with only semi-mesenchymal CTCs. This is a very interesting finding and may indicate that there is also a subgroup of ovarian cancer patients with “fully” epithelial CTCs, without any shifts towards EMT. These CTCs may have entered the bloodstream via passive dissemination [50], however, clinical relevance of this finding requires further investigation.

Applying our EMT-associated marker set, we revealed a heterogeneous spectrum of EMT-associated CTC-phenotypes, pointing to CTC-heterogeneity in ovarian cancer, which has already been reported for CTCs in a variety of other cancer entities, such as prostate or breast cancer [51, 52]. Notably, we cannot distinguish with our assay, whether a co-expression of more than one marker-transcript is derived from CTCs, actually co-expressing these markers on a same cell, or from separate semi-mesenchymal CTC-populations, concomitantly present in the “pool” of enriched CTCs.

Oncogenic EMT has received considerable attention over the past years and is associated with cancer aggressiveness, metastasis and tumor cell plasticity [29]. Interestingly, we observed a clear increase of semi-mesenchymal CTCs in response to platinum-based chemotherapy, due to a shift towards PI3Kα and Twist expression. This finding is of particular clinical interest, since it has already been described that cancers may acquire resistance to systemic treatment as a result of clonal evolution and selection [53]. We therefore assume a clonal selection of CTCs with activated PI3Kα and Twist associated signaling pathways, which might be therapy refractory and could be responsible for recurrence of ovarian cancer. This assumption is strongly supported by our previous studies on breast cancer patients, in which we already showed that preferentially semi-mesenchymal and potentially platinum-resistant (ERCC1-expressing) CTCs remain after neoadjuvant chemotherapy [54]. Moreover, a further independent key publication reported on dynamic changes in the epithelial and mesenchymal composition of breast cancer CTCs in response to therapy. In this study, clinical response was accompanied by a switch to predominantly epithelial CTCs, whereas progressive disease correlated with the increase of mesenchymal CTC-phenotypes [21]. For ovarian cancer, *in vitro* experiments already suggested that cisplatin treatment of ovarian cancer cells generates residual cells with EMT-like traits [36]. Therefore, we assume that PI3Kα and Twist positive CTCs may reflect tumor evolution in response to platinum-based chemotherapy. Interestingly, this also suggests a link to recent studies on genomic tumor evolution, reporting on an increase in activating PIK3CA mutation among cell free tumor DNA of breast cancer patients, following paclitaxel treatment [53]. In this context, our findings could have several diagnostic or therapeutic implications, since PI3Kα and Twist are functionally involved in pathways controlling tumor cell survival or platinum-resistance [55, 56]. The most important limitation of our study is the small number of patients, particularly when comparing the detection frequency of single transcript markers in pre- and post-therapeutic blood samples. Nevertheless, this detailed analysis, albeit only descriptive, was considerably informative for us and complemented our key finding, the enrichment of semi-mesenchymal CTC after platinum-based chemotherapy.

Among our exploratory survival analysis, we did not confirm prognostic relevance of epithelial CTCs in the unselected study population, which is in contrast to our previous finding and could be explained by the yet limited follow-up period of the present study [10]. Nevertheless, after excluding FIGO IV patients as possible confounders, prognostic relevance of epithelial CTCs could be restored. Apart from this, the presence of epithelial CTCs at primary diagnosis, in combination with PI3Kα-positivity, indicated poor prognosis not only in FIGO I-III patients, but also in the unselected cohort, suggesting that PI3Kα-positivity marks a clinically relevant subgroup of EMT-associated CTCs, which could be derived from the untreated primary tumor. In contrast, we reported that patients with residual tumor after primary debulking were more likely to be positive for EMT-like CTCs after adjuvant chemotherapy, indicating that this CTC-population could be disseminated directly from residual tumor burden under the selective pressure of chemotherapy, after conversion to a semi-mesenchymal state.

Conclusively, this is the first study on EMT-like CTCs in ovarian cancer, reporting that platinum-based chemotherapy provokes a shift of molecular phenotypes towards PI3Kα and Twist expressing CTCs, which may reflect clonal tumor evolution. Therefore, we encourage to further investigate the functional role of semi-mesenchymal CTCs in the malignant progression of ovarian cancer and to determine, whether these EMT-like CTCs can be a biomarker for high risk minimal residual disease in ovarian cancer. In this context, an extended multi-marker panel with mesenchymal and tumor stem cell-associated genes, which we recently established for metastatic breast cancer [18], could be a useful liquid biopsy tool, in order to detect a broad spectrum of CTC-phenotypes for therapy monitoring. Moreover, PI3Kα and Twist positive CTCs could be an attractive therapeutic target, since PI3K/Akt/mTOR pathway inhibitors are currently being investigated for ovarian cancer among several preclinical studies and also a few ongoing clinical trials [32] (NCT01623349, NCT02476955). Moreover, it was shown for ovarian cancer that low dose metformin, a first line drug for treating diabetes, can also reduce the expression of EMT-associated makers, such as Twist, suggesting further options for potentially targeting this CTC-population [57]. Since the biology of ovarian cancer CTCs is largely unknown, we believe that our finding could be a step forward in understanding their heterogeneity and their dynamics in response to chemotherapy.

## MATERIALS AND METHODS

### Patient characteristics

The present study was conducted at the Departments of Gynecology and Obstetrics at the University Hospitals of Essen and Dresden, Germany. In this study, a total of 95 patients, diagnosed between 2010 and 2014 with histologically confirmed epithelial ovarian cancer, were analyzed. Clinical characteristics of the patients are documented in Table 1. Informed written consent was obtained from all patients and the study was approved by the Local Ethic Committees (Essen 05–2870; Dresden EK 236082012) and was performed according to the declaration of Helsinki. Tumors were classified according to the WHO classification of tumors of the female genital tract. Grading was conducted using the grading system proposed by Silverberg [58] and tumor staging was classified according to the Fédération Internationale de Gynécology et d'Obstétrique. The whole study population underwent primary radical surgery. Total abdominal hysterectomy, bilateral salpingo-oophorectomy, infragastric omentectomy and peritoneal stripping was performed. The most important aim of surgery was to achieve macroscopic complete tumor resection. Radical pelvic and para-aortic lymphadenectomy were only performed if macroscopic complete tumor resection was achieved. All patients received at least six cycles of carboplatinum AUC 5 and paclitaxel 175 mg/m^2^.

**Table 1:** Patient characteristics at the time of primary diagnosis

Total	95
Age	median 61 years, (31–82) years
FIGO stage	
I–II	16 (17%)
III	61 (64%)
IV	18 (19%)
Nodal status	
No	33 (37%)
N1	32 (43%)
Nx	30 (20%)
Grading	
I–II	32 (43%)
III	63 (57%)
Residual tumor	
Macroscopic complete resection	48 (51%)
Any residual tumor	40 (42%)
Unknown	7 (7%)
Histologic type	
Serous	81 (80%)
Mucinous	5 (14%)
Other	9 (6%)
Survival	
PFS 1	median 6 months, (0–51 months)
OS2	median 21 months, (1–59 months)
Alive	15 (16%)
Dead	72 (76%)
Unknown	8 (8%)
Recurrence	
No relapse	50 (43%)
Relapse	28 (55%)
Unknown	17 (2%)

^1^PFS: progression-free survival, ^2^OS: overall survival

### Enrichment and molecular characterization of ovarian cancer CTCs

Peripheral blood (5 ml) from each patient was collected in EDTA tubes (Sarstedt, Nümbrecht, Germany) and processed within 4h for the enrichment of CTCs and subsequent expression analysis, according to the AdnaTest *OvarianCancer Detect* and the AdnaTest *EMT-1 Detect* (QIAGEN, Hilden, Langenhagen, Germany, Supplementary Figure 1, 2). These assays have been described in detail elsewhere [10, 15, 44]. The Adnatest was performed in biological replicates; therefore two independent consecutive blood samples were obtained from each patient at each time point. Briefly, we applied immuno-magnetical enrichment of CTCs (Adnatest *OvarianCancer Select*, Adnatest *EMT-1 Select)*, targeting epithelial cellular adhesion molecules.

For the detection of epithelial CTCs, RNA was isolated and gene expression analysis was performed by reverse-transcription (RT) and multiplex RT-PCR, detecting EpCAM, Muc-1, and CA-125 (AdnaTest *OvarianCancer Detect)*. In this assay, amplicons with the following sizes were generated: EpCAM: 396bp; Muc-1: 293bp; CA-125: 432bp. For the detection EMT-like CTCs, RNA was isolated and gene expression analysis was performed by reverse-transcription (RT) and multiplex RT-PCR, detecting PI3Kα, Akt-2 and Twist (AdnaTest *EMT-1 Detect)*. Contaminating leukocytes (about 1500 per sample) were reduced by approximately 10fold using a special washing buffer (*AdnaWash* buffer) enabling the proper differentiation of EMT expression profiles with a specificity and sensitivity of > 90%, which was confirmed in healthy donor samples [20, 44]. In this assay, amplicons with the following sizes were generated: PI3Kα: 595bp, Akt-2: 306 bp; Twist: 203bp. β-actin served as an internal control (amplicon size: 119 bp) and PCR-products were visualized with the Agilent Bioanalyzer (Agilent Technologies, Santa Clara, USA). An amplicon concentration of > 0.2 ng/μl was applied as threshold for EpCAM, Muc-1 or CA-125 positivity. Amplicon concentration of > 0.2 ng/μl was applied as threshold for Akt-2, > 0.25 ng/μl for PI3Kα and > 0,15 ng/μl for Twist positivity, respectively.

The analytical sensitivity of the detection of CTC-associated EMT-transcripts was determined by the analysis of a low number of target cells (5 IGROV ovarian cancer cells spiked into 5 ml blood of healthy donors). Healthy donor samples without spiked tumor cells were used to determine the specificity of the test. Applying the above mentioned amplicon cut-off values, 97% of 30 healthy donor samples were negative for EMT markers. These experiments demonstrate that a potential risk of false-positive events is negligible in our present analysis.

## SUPPLEMENTARY MATERIALS FIGURES AND TABLE



## Abbreviations

BM: bone marrow
CTC(s): circulating tumor cell(s)
DTC(s): disseminated tumor cell(s)
EMT: epithelial-to-mesenchymal-transition
EpCAM: epithelial cell adhesion molecule
ERCC1: excision repair cross-complementation group 1
MET: mesenchymal-to-epithelial-transition
Muc-1: Mucin-1
OS: overall survial
PFS: progression free survival
RT: reverse transcription

## References

[1] Goodman MT, Howe HL, Tung KH, Hotes J, Miller BA, Coughlin SS, Chen VW (2003). Incidence of ovarian cancer by race and ethnicity in the United States, 1992–1997. Cancer.

[2] du Bois A, Quinn M, Thigpen T, Vermorken J, Avall-Lundqvist E, Bookman M, Bowtell D, Brady M, Casado A, Cervantes A, Eisenhauer E, Friedlaender M, Fujiwara K (2005). 2004 consensus statements on the management of ovarian cancer: final document of the 3rd International Gynecologic Cancer Intergroup Ovarian Cancer Consensus Conference (GCIG OCCC 2004). Ann Oncol.

[3] du Bois A, Reuss A, Pujade-Lauraine E, Harter P, Ray-Coquard I, Pfisterer J (2009). Role of surgical outcome as prognostic factor in advanced epithelial ovarian cancer: a combined exploratory analysis of 3 prospectively randomized phase 3 multicenter trials: by the Arbeitsgemeinschaft Gynaekologische Onkologie Studiengruppe Ovarialkarzinom (AGO-OVAR) and the Groupe d'Investigateurs Nationaux Pour les Etudes des Cancers de l'Ovaire (GINECO). Cancer.

[4] Wimberger P, Lehmann N, Kimmig R, Burges A, Meier W, Du Bois A, Arbeitsgemeinschaft Gynaekologische Onkologie Ovarian Cancer Study G (2007). Prognostic factors for complete debulking in advanced ovarian cancer and its impact on survival. An exploratory analysis of a prospectively randomized phase III study of the Arbeitsgemeinschaft Gynaekologische Onkologie Ovarian Cancer Study Group (AGO-OVAR). Gynecol Oncol.

[5] Wimberger P, Wehling M, Lehmann N, Kimmig R, Schmalfeldt B, Burges A, Harter P, Pfisterer J, du Bois A (2010). Influence of residual tumor on outcome in ovarian cancer patients with FIGO stage IV disease: an exploratory analysis of the AGO-OVAR (Arbeitsgemeinschaft Gynaekologische Onkologie Ovarian Cancer Study Group). Annals of Surgical Oncology.

[6] Martin LP, Schilder RJ (2009). Management of recurrent ovarian carcinoma: current status and future directions. Seminars in Oncology.

[7] Burger RA, Brady MF, Bookman MA, Fleming GF, Monk BJ, Huang H, Mannel RS, Homesley HD, Fowler J, Greer BE, Boente M, Birrer MJ, Liang SX (2011). Incorporation of bevacizumab in the primary treatment of ovarian cancer. N Engl J Med.

[8] Ledermann J, Harter P, Gourley C, Friedlander M, Vergote I, Rustin G, Scott CL, Meier W, Shapira-Frommer R, Safra T, Matei D, Fielding A, Spencer S (2014). Olaparib maintenance therapy in patients with platinum-sensitive relapsed serous ovarian cancer: a preplanned retrospective analysis of outcomes by BRCA status in a randomised phase 2 trial. Lancet Oncol.

[9] Romero-Laorden N, Olmos D, Fehm T, Garcia-Donas J, Diaz-Padilla I (2014). Circulating and disseminated tumor cells in ovarian cancer: a systematic review. Gynecol Oncol.

[10] Aktas B, Kasimir-Bauer S, Heubner M, Kimmig R, Wimberger P (2011). Molecular profiling and prognostic relevance of circulating tumor cells in the blood of ovarian cancer patients at primary diagnosis and after platinum-based chemotherapy. Int J Gynecol Cancer.

[11] Obermayr E, Castillo-Tong DC, Pils D, Speiser P, Braic I, Van Gorp T, Mahner S, Sehouli J, Vergote I, Zeillinger R (2013). Molecular characterization of circulating tumor cells in patients with ovarian cancer improves their prognostic significance—a study of the OVCAD consortium. Gynecol Oncol.

[12] Wimberger P, Heubner M, Otterbach F, Fehm T, Kimmig R, Kasimir-Bauer S (2007). Influence of platinum-based chemotherapy on disseminated tumor cells in blood and bone marrow of patients with ovarian cancer. Gynecol Oncol.

[13] Fan T, Zhao Q, Chen JJ, Chen WT, Pearl ML (2009). Clinical significance of circulating tumor cells detected by an invasion assay in peripheral blood of patients with ovarian cancer. Gynecol Oncol.

[14] Zhou Y, Bian B, Yuan X, Xie G, Ma Y, Shen L (2015). Prognostic Value of Circulating Tumor Cells in Ovarian Cancer: A Meta-Analysis. PLoS One.

[15] Kuhlmann JD, Wimberger P, Bankfalvi A, Keller T, Scholer S, Aktas B, Buderath P, Hauch S, Otterbach F, Kimmig R, Kasimir-Bauer S (2014). ERCC1-positive circulating tumor cells in the blood of ovarian cancer patients as a predictive biomarker for platinum resistance. Clin Chem.

[16] Chebouti I, Blassl C, Wimberger P, Neubauer H, Fehm T, Kimmig R, Kasimir-Bauer S (2016). Analysis of disseminated tumor cells before and after platinum based chemotherapy in primary ovarian cancer. Do stem cell like cells predict prognosis?. Oncotarget.

[17] Blassl C, Kuhlmann JD, Webers A, Wimberger P, Fehm T, Neubauer H (2016). Gene expression profiling of single circulating tumor cells in ovarian cancer - Establishment of a multi-marker gene panel. Mol Oncol.

[18] Bredemeier M, Edimiris P, Tewes M, Mach P, Aktas B, Schellbach D, Wagner J, Kimmig R, Kasimir-Bauer S (2016). Establishment of a multimarker qPCR panel for the molecular characterization of circulating tumor cells in blood samples of metastatic breast cancer patients during the course of palliative treatment. Oncotarget.

[19] Brouwer A, De Laere B, Peeters D, Peeters M, Salgado R, Dirix L, Van Laere S (2016). Evaluation and consequences of heterogeneity in the circulating tumor cell compartment. Oncotarget.

[20] Aktas B, Tewes M, Fehm T, Hauch S, Kimmig R, Kasimir-Bauer S (2009). Stem cell and epithelial-mesenchymal transition markers are frequently overexpressed in circulating tumor cells of metastatic breast cancer patients. Breast Cancer Res.

[21] Yu M, Bardia A, Wittner BS, Stott SL, Smas ME, Ting DT, Isakoff SJ, Ciciliano JC, Wells MN, Shah AM, Concannon KF, Donaldson MC, Sequist LV (2013). Circulating breast tumor cells exhibit dynamic changes in epithelial and mesenchymal composition. Science.

[22] Liu H, Zhang X, Li J, Sun B, Qian H, Yin Z (2014). The biological and clinical importance of epithelial-mesenchymal transition in circulating tumor cells. J Clin Med.

[23] Shih JY, Tsai MF, Chang TH, Chang YL, Yuan A, Yu CJ, Lin SB, Liou GY, Lee ML, Chen JJ, Hong TM, Yang SC, Su JL (2005). Transcription repressor slug promotes carcinoma invasion and predicts outcome of patients with lung adenocarcinoma. Clin Cancer Res.

[24] Drake JM, Strohbehn G, Bair TB, Moreland JG, Henry MD (2009). ZEB1 enhances transendothelial migration and represses the epithelial phenotype of prostate cancer cells. Mol Biol Cell.

[25] Smit MA, Geiger TR, Song JY, Gitelman I, Peeper DS (2009). A Twist-Snail axis critical for TrkB-induced epithelial-mesenchymal transition-like transformation, anoikis resistance, and metastasis. Mol Cell Biol.

[26] Stoletov K, Kato H, Zardouzian E, Kelber J, Yang J, Shattil S, Klemke R (2010). Visualizing extravasation dynamics of metastatic tumor cells. J Cell Sci.

[27] Ocana OH, Corcoles R, Fabra A, Moreno-Bueno G, Acloque H, Vega S, Barrallo-Gimeno A, Cano A, Nieto MA (2012). Metastatic colonization requires the repression of the epithelial-mesenchymal transition inducer Prrx1. Cancer Cell.

[28] Jiang J, Tang YL, Liang XH (2011). EMT: a new vision of hypoxia promoting cancer progression. Cancer Biol Ther.

[29] Kalluri R, Weinberg RA (2009). The basics of epithelial-mesenchymal transition. J Clin Invest.

[30] Levine DA, Bogomolniy F, Yee CJ, Lash A, Barakat RR, Borgen PI, Boyd J (2005). Frequent mutation of the PIK3CA gene in ovarian and breast cancers. Clin Cancer Res.

[31] Engelman JA, Luo J, Cantley LC (2006). The evolution of phosphatidylinositol 3-kinases as regulators of growth and metabolism. Nat Rev Genet.

[32] Liu P, Cheng H, Roberts TM, Zhao JJ (2009). Targeting the phosphoinositide 3-kinase pathway in cancer. Nat Rev Drug Discov.

[33] Xu W, Yang Z, Lu N (2015). A new role for the PI3K/Akt signaling pathway in the epithelial-mesenchymal transition. Cell Adhesion & Migration.

[34] Tania M, Khan MA, Fu J (2014). Epithelial to mesenchymal transition inducing transcription factors and metastatic cancer. Tumour Biology.

[35] Ahmed N, Abubaker K, Findlay J, Quinn M (2010). Epithelial mesenchymal transition and cancer stem cell-like phenotypes facilitate chemoresistance in recurrent ovarian cancer. Curr Cancer Drug Targets.

[36] Latifi A, Abubaker K, Castrechini N, Ward AC, Liongue C, Dobill F, Kumar J, Thompson EW, Quinn MA, Findlay JK, Ahmed N (2011). Cisplatin treatment of primary and metastatic epithelial ovarian carcinomas generates residual cells with mesenchymal stem cell-like profile. J Cell Biochem.

[37] Davidson B, Trope CG, Reich R (2012). Epithelial-mesenchymal transition in ovarian carcinoma. Front Oncol.

[38] Lianidou ES, Markou A (2011). Circulating tumor cells in breast cancer: detection systems, molecular characterization, and future challenges. Clin Chem.

[39] Joosse SA, Gorges TM, Pantel K (2015). Biology, detection, and clinical implications of circulating tumor cells. EMBO Mol Med.

[40] Xenidis N, Ignatiadis M, Apostolaki S, Perraki M, Kalbakis K, Agelaki S, Stathopoulos EN, Chlouverakis G, Lianidou E, Kakolyris S, Georgoulias V, Mavroudis D (2009). Cytokeratin-19 mRNA-positive circulating tumor cells after adjuvant chemotherapy in patients with early breast cancer. J Clin Oncol.

[41] Heitzer E, Auer M, Gasch C, Pichler M, Ulz P, Hoffmann EM, Lax S, Waldispuehl-Geigl J, Mauermann O, Lackner C, Hofler G, Eisner F, Sill H (2013). Complex tumor genomes inferred from single circulating tumor cells by array-CGH and next-generation sequencing. Cancer Res.

[42] Xenidis N, Perraki M, Kafousi M, Apostolaki S, Bolonaki I, Stathopoulou A, Kalbakis K, Androulakis N, Kouroussis C, Pallis T, Christophylakis C, Argyraki K, Lianidou ES (2006). Predictive and prognostic value of peripheral blood cytokeratin-19 mRNA-positive cells detected by real-time polymerase chain reaction in node-negative breast cancer patients. J Clin Oncol.

[43] Andreopoulou E, Yang LY, Rangel KM, Reuben JM, Hsu L, Krishnamurthy S, Valero V, Fritsche HA, Cristofanilli M (2012). Comparison of assay methods for detection of circulating tumor cells in metastatic breast cancer: AdnaGen AdnaTest BreastCancer Select/Detect versus Veridex CellSearch system. Int J Cancer.

[44] Kasimir-Bauer S, Hoffmann O, Wallwiener D, Kimmig R, Fehm T (2012). Expression of stem cell and epithelial-mesenchymal transition markers in primary breast cancer patients with circulating tumor cells. Breast Cancer Res.

[45] Rhim AD, Mirek ET, Aiello NM, Maitra A, Bailey JM, McAllister F, Reichert M, Beatty GL, Rustgi AK, Vonderheide RH, Leach SD, Stanger BZ (2012). EMT and dissemination precede pancreatic tumor formation. Cell.

[46] Pascal LE, True LD, Campbell DS, Deutsch EW, Risk M, Coleman IM, Eichner LJ, Nelson PS, Liu AY (2008). Correlation of mRNA and protein levels: cell type-specific gene expression of cluster designation antigens in the prostate. BMC Genomics.

[47] Greenbaum D, Colangelo C, Williams K, Gerstein M (2003). Comparing protein abundance and mRNA expression levels on a genomic scale. Genome Biol.

[48] Nie L, Wu G, Zhang W (2006). Correlation of mRNA expression and protein abundance affected by multiple sequence features related to translational efficiency in Desulfovibrio vulgaris: a quantitative analysis. Genetics.

[49] Bednarz-Knoll N, Alix-Panabieres C, Pantel K (2012). Plasticity of disseminating cancer cells in patients with epithelial malignancies. Cancer Metastasis Rev.

[50] Alpaugh ML, Tomlinson JS, Kasraeian S, Barsky SH (2002). Cooperative role of E-cadherin and sialyl-Lewis X/A-deficient MUC1 in the passive dissemination of tumor emboli in inflammatory breast carcinoma. Oncogene.

[51] Massard C, Oulhen M, Le Moulec S, Auger N, Foulon S, Abou-Lovergne A, Billiot F, Valent A, Marty V, Loriot Y, Fizazi K, Vielh P, Farace F (2016). Phenotypic and genetic heterogeneity of tumor tissue and circulating tumor cells in patients with metastatic castrationresistant prostate cancer: a report from the PETRUS prospective study. Oncotarget.

[52] Shaw JA, Guttery DS, Hills A, Fernandez-Garcia D, Page K, Rosales BM, Goddard KS, Hastings RK, Luo J, Ogle O, Woodley L, Ali S, Stebbing J (2016). Mutation analysis of cell-free DNA and single circulating tumor cells in metastatic breast cancer patients with high CTC counts. Clin Cancer Res.

[53] Murtaza M, Dawson SJ, Tsui DW, Gale D, Forshew T, Piskorz AM, Parkinson C, Chin SF, Kingsbury Z, Wong AS, Marass F, Humphray S, Hadfield J (2013). Non-invasive analysis of acquired resistance to cancer therapy by sequencing of plasma DNA. Nature.

[54] Kasimir-Bauer S, Bittner AK, Konig L, Reiter K, Keller T, Kimmig R, Hoffmann O (2016). Does primary neoadjuvant systemic therapy eradicate minimal residual disease? Analysis of disseminated and circulating tumor cells before and after therapy. Breast Cancer Res.

[55] Kolasa IK, Rembiszewska A, Felisiak A, Ziolkowska-Seta I, Murawska M, Moes J, Timorek A, Dansonka-Mieszkowska A, Kupryjanczyk J (2009). PIK3CA amplification associates with resistance to chemotherapy in ovarian cancer patients. Cancer Bio Ther.

[56] Nuti SV, Mor G, Li P, Yin G (2014). TWIST and ovarian cancer stem cells: implications for chemoresistance and metastasis. Oncotarget.

[57] Zhang R, Zhang P, Wang H, Hou D, Li W, Xiao G, Li C (2015). Inhibitory effects of metformin at low concentration on epithelial-mesenchymal transition of CD44(+)CD117(+) ovarian cancer stem cells. Stem Cell Res.

[58] Silverberg SG (2000). Histopathologic grading of ovarian carcinoma: a review and proposal. Int J Gynecol Pathol.

