# Supplementary material for: EMT-like circulating tumor cells in ovarian cancer patients are enriched by platinum-based chemotherapy
Source: Oncotarget. 2017 Mar 14;8(30):48820–31. doi: 10.18632/oncotarget.16179 (PMC5564727; doi:10.18632/oncotarget.16179)
Supplement: Supplementary file 1 [file oncotarget-08-48820-s001.pdf]

EMT-like circulating tumor cells in ovarian cancer patients are enriched by platinum-based chemotherapy

SUPPLEMENTARY MATERIALS

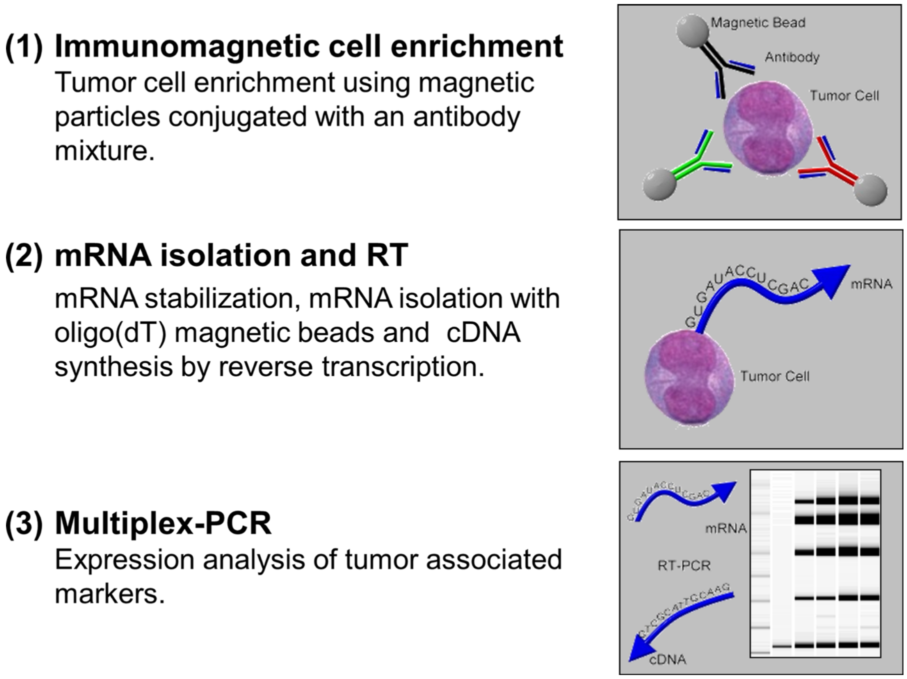

Supplementary Figure 1: Principle of the Adnatest *OvarianCancer*. The figure shows the different steps, how CTCs are detected by the Adnatest OvarianCancer from whole blood. CTCs are immunomagnetically enriched via magnetic beads, which are coated with antibodies that bind to epithelial CTC-epitopes. Subsequently, RNA is isolated and gene expression analysis is performed by multiplex RT-PCR.

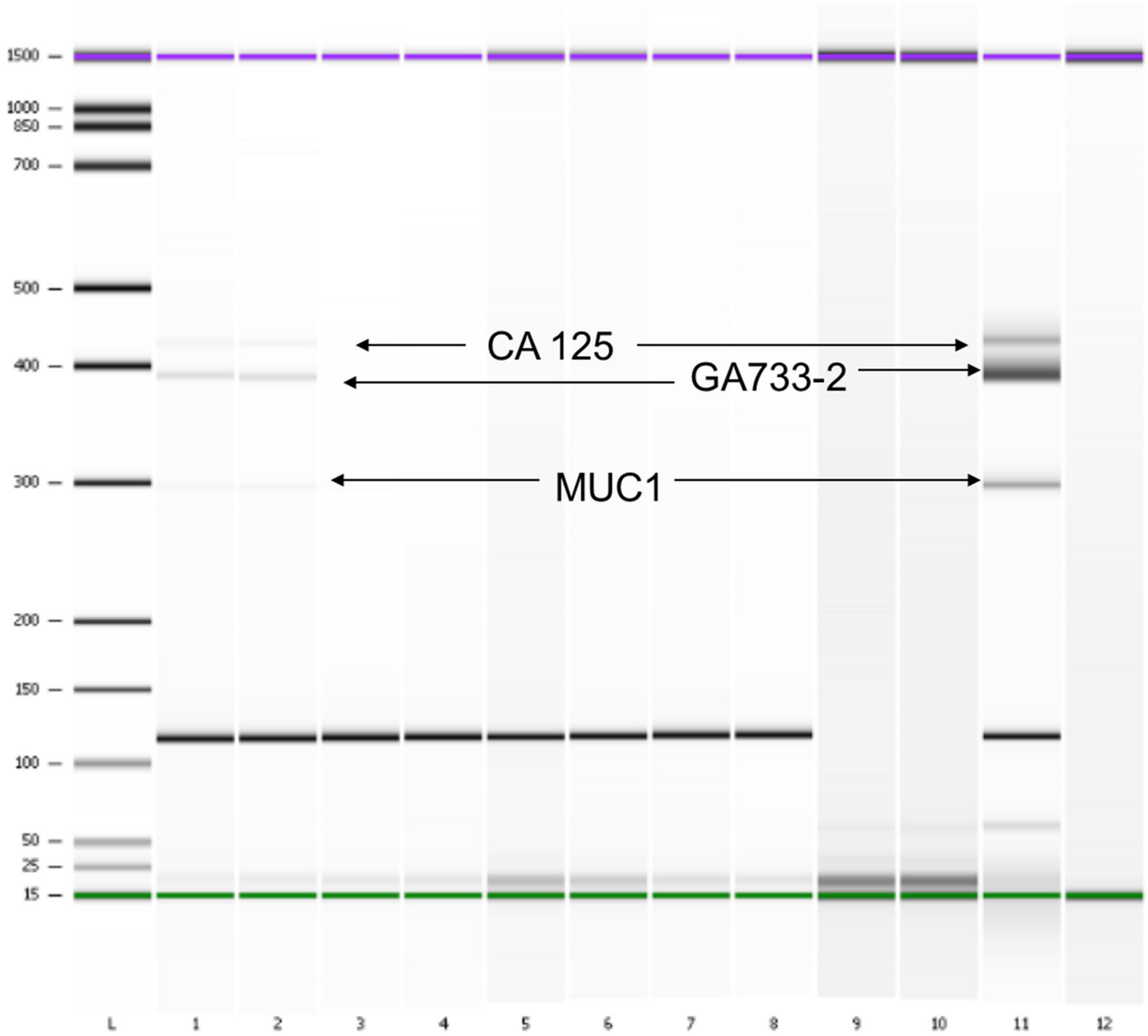

Supplementary Figure 2: Analysis of transcript markers of the Adnatest *OvarianCancer*. The figure shows a representative electropherogram of CTC-analysis in an ovarian cancer patient, which is positive for epithelial CTC-associated transcript markers. Amplicons with the following sizes were generated: EpCAM: 396bp; Muc-1: 293bp, CA-125: 432bp.

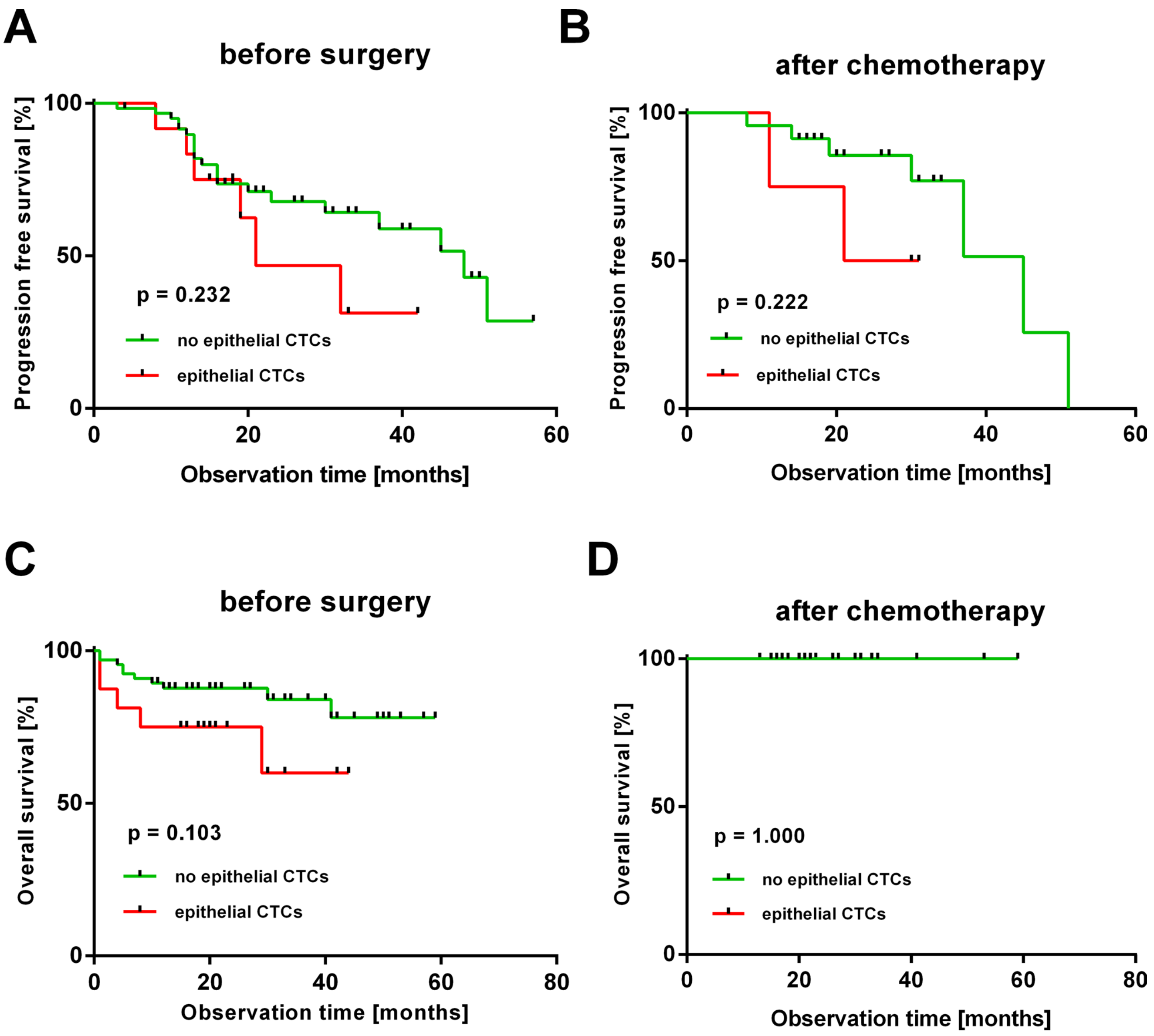

Supplementary Figure 3: Prognostic relevance of epithelial CTCs. The figure shows Kaplan-Meier analysis, comparing progression-free survival (PFS, A+B) or overall survival (OS, C+D) of patients with the presence of epithelial CTCs in their blood (red curves) vs. patients without epithelial CTCs (green curves). There was no statistical significance in survival between the compared patient groups.

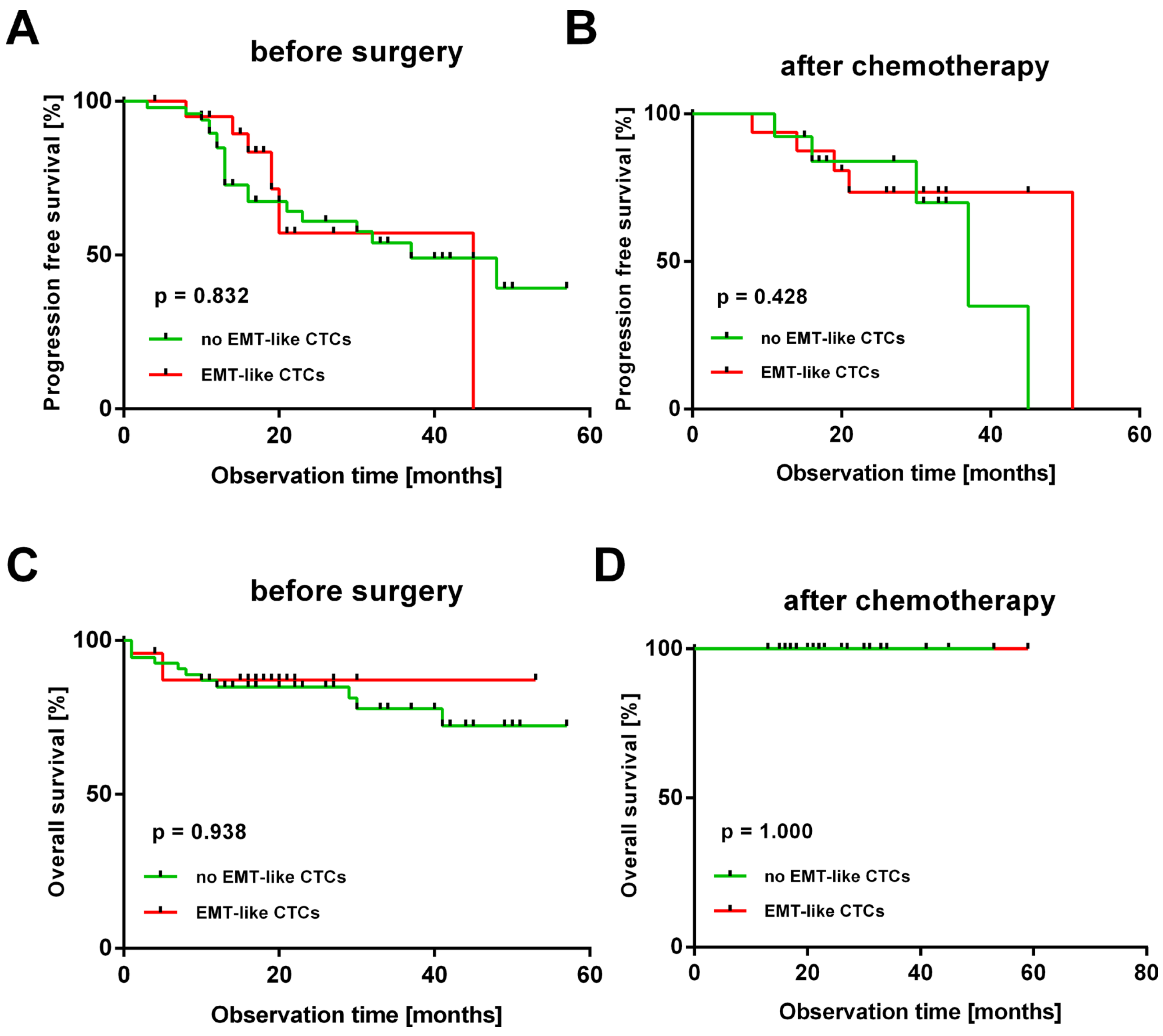

Supplementary Figure 4: Prognostic relevance of EMT-like CTCs. The figure shows Kaplan-Meier analysis, comparing progression-free survival (PFS, A+B) or overall survival (OS, C+D) of patients with the presence of EMT-like CTCs in their blood (red curves) vs. patients without EMT-like CTCs (green curves). There was no statistical significance in survival between the compared patient groups.

Supplementary Table 1: Distribution of epithelial and EMT-associated transcripts in CTCs of ovarian cancer patients

Before Surgery				After Chemotherapy			
**Figure 1**	**Number of positive patients**	***n***	**%**		**Number of positive patients**	***n***	**%**
epithelial CTCs	16	91	**18**	epithelial CTCs	4	29	**14**
EMT-like CTCs	26	86	**30**	EMT-like CTCs	16	31	**52**
**Figure 2**							
epithelial CTC-positive/ EMT-like CTC-positive	6	34	**18**	epithelial CTC-positive/ EMT-like CTC-positive	2	17	**12**
epithelial CTC-positive/ EMT-like CTC-negative	8	34	**24**	epithelial CTC-positive/ EMT-like CTC-negative	2	17	**12**
epithelial CTC-negative/ EMT-like CTC-positive	20	34	**59**	epithelial CTC-negative/ EMT-like CTC-positive	13	17	**76**
**Figure 3a**							
EpCAM	6	16	**38**	EpCAM	1	4	**25**
Muc-1	13	16	**81**	Muc-1	3	4	**75**
**Figure 3b**							
EpCAM	3	16	**19**	EpCAM	1	4	**25**
Muc-1	10	16	**63**	Muc-1	3	4	**75**
**Figure 3c**							
PI3Kα	9	26	**35**	PI3Kα	5	16	**31**
AKT	12	26	**46**	AKT	6	16	**38**
Twist	14	26	**54**	Twist	11	16	**69**
**Figure 3d**							
PI3Kα	3	26	**12**	PI3Kα	1	16	**6**
AKT	5	26	**19**	AKT	3	16	**19**
Twist	11	26	**42**	Twist	7	16	**44**
PI3Kα +Akt-2	4	26	**15**	PI3Kα +Akt-2	1	16	**6**
PI3Kα +Twist	0	26	**0**	PI3Kα +Twist	2	16	**13**
Akt-2+Twist	1	26	**4**	Akt-2+Twist	1	16	**6**
PI3Kα +Akt-2+Twist	2	26	**8**	PI3Kα +Akt-2+Twist	1	16	**6**

The table summarizes the absolute counts and calculated positivity rates of patients with different CTC-subtypes, according to epithelial and EMT-associated CTCs. CTC-subtypes are listed as they chronologically appear in Figure 1-3 in the manuscript. According to Figure 1, a patient was counted positive for epithelial or EMT-like CTCs, as soon as at least one of the epithelial or EMT-associated markers was detected in one of the two biological replicate samples. According to Figure 2, co-incidence of positivity for epithelial and EMT-like CTCs was assessed. In Figure 3a and Figure 3c, percentages for (EpCAM or Muc-1) or (PI3Kα or Akt-2 or Twist) were calculated, independently from each other, and in reference to only those patients with positivity for epithelial or EMT-like CTCs, respectively. In Figure 3b and 3d, percentages were calculated in reference to patients with positivity for epithelial or EMT-like CTCs, respectively, also considering dual or triple marker positivity.
